# The effects of crossbow impacts onto a common automotive vehicle side window—a preliminary study

**DOI:** 10.1007/s00414-019-02171-5

**Published:** 2019-11-07

**Authors:** R. Critchley, K. Standbridge, A. Peare

**Affiliations:** Centre for Defence Engineering, Cranfield University, Defence Academy of the United Kingdom, Shrivenham, SN6 8LA UK

**Keywords:** Crossbow, Impact, Glass, Automotive Glass, Bolts

## Abstract

In recent times, the number of criminal incidents involving crossbows in the UK has increased with many incidents resulting in either injuries or fatalities. Whilst the effects of crossbow bolts on the body are well understood, there is a limited understanding on how these projectiles interact with the wider environment. One area of particular interest is the interaction between common vehicle side windows and bolts. In this study, the penetrability of two distinct bolts using an off-the-shelve crossbow against a common automotive side window was explored, where velocity loss up to 25 m/s post impact was recorded. All windows failed through radial glass fracture at a rate up to 1600 m/s, whilst bolt damage varied from tip holder decoupling, shaft damage, and traumatic fletching removal. No distinct relationship between bolt type, velocity, and window damage was identified.

## Introduction

In recent years the number of criminal incidents involving crossbows in the UK has become more apparent, with many incidents resulting in either injuries or fatalities [1–5]. Whilst the apparent increase of these incidents could potentially be attributed to media reporting bias, their reporting in academic literature has risen over the last few years [6–20]. Although the majority of incidents involving injury occur within open environments, there is evidence that individuals within vehicles are at risk [21, 22]. In these incidents it was evident that the windows of the vehicle failed to arrest the crossbow bolt and that the lack of physical injury could be considered fortunate.

Under the UK law, crossbows are not considered firearms whilst their ownership is restricted to over eighteen years old by the Crossbows Act 1987 and Crossbows Order 1998 (Northern Ireland) [23]. The UK’s legislation towards crossbow registration is non-existent and thus the total number of crossbows held within the UK is unknown. As of 1986 [24], it was approximated that 200,000 crossbows were owned; a number suspected to now be much greater.

Unlike their historical counterparts, today’s crossbows are typically used for sport shooting within the UK. The bow is constructed with a horizontal limb formation; the limbs are mounted to a stock. The crossbow has the capability to fire quarrels or bolts. However, crossbows have also been designed to shoot rocks and other projectiles [25]. Under the guide line of hunting crossbows, a crossbow cannot be produced with a draw weight exceeding 290 lbs, which under the right conditions can deliver up to 173 J [26]. For comparison, a Glock pistol and an AK47 have approximate muzzle energies up to 730 J and 3200 J depending on ammunition used, respectively [27].

Whilst such energy levels are below common firearms, crossbows still remain a valid threat due to their ease of procurement and potential lethality. As such understanding not only how crossbow bolts interact with the body but with the environment is of paramount importance for the forensic examiner. One area of particular interest that suffers from porosity within the literature is the interaction between common vehicle side windows and bolts and their potential source of forensic information. As such this paper explores the penetrability of an off-the-shelve crossbow and bolt combinations against a common automotive side window. The effect of impact velocity is also explored with findings discussed in terms of forensic evidence and what it means for the investigators.

## Materials and methods

### Crossbow and projectiles

A Jaguar II crossbow of draw weight 175 lb was purchased, along with bolts (2219 hybrid carbon bolt and EK Archery research bolt). Bolt details are given at [28, 29] and a scope to represent a typical system and ammunition that an attacker may use. The system is considered to be mid-range crossbow and is capable of delivering bolts at a velocity of 260 fps (79 m/s) [30].

### Targets

**Fig. 1:**
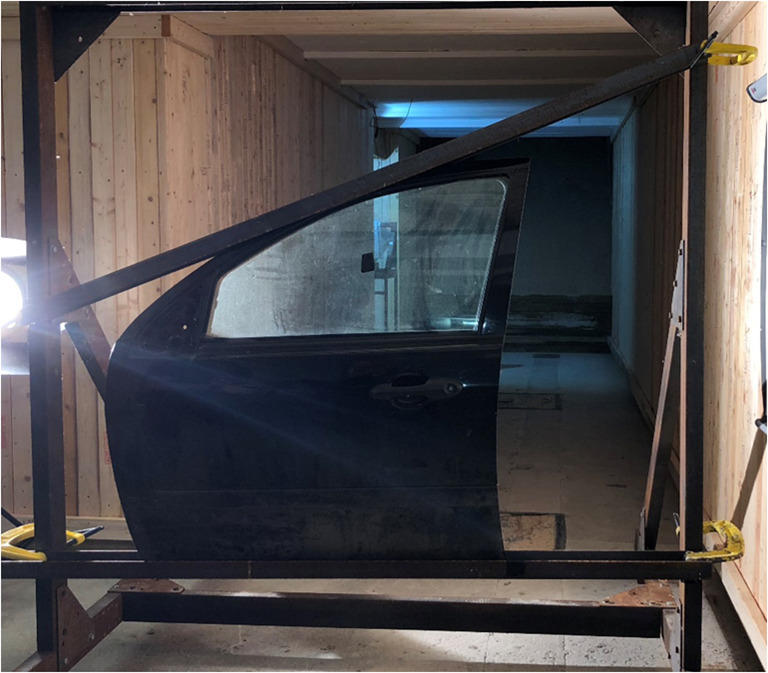
Target door secured in mount

Six Ford Fiesta (Fifth generation model, year 2002 to 2008) front doors containing tempered glass were procured from Haynes of Challow, Oxfordshire to represent a common car type within the UK. Prior to use, each door window was visibly checked for damage before being hand cleaned with soap and warm water to remove any visible dirt or blemishes before placed in a metal target frame (Fig. 1). Doors were positioned with the external face facing towards the crossbow.

### Setup

**Fig. 2:**
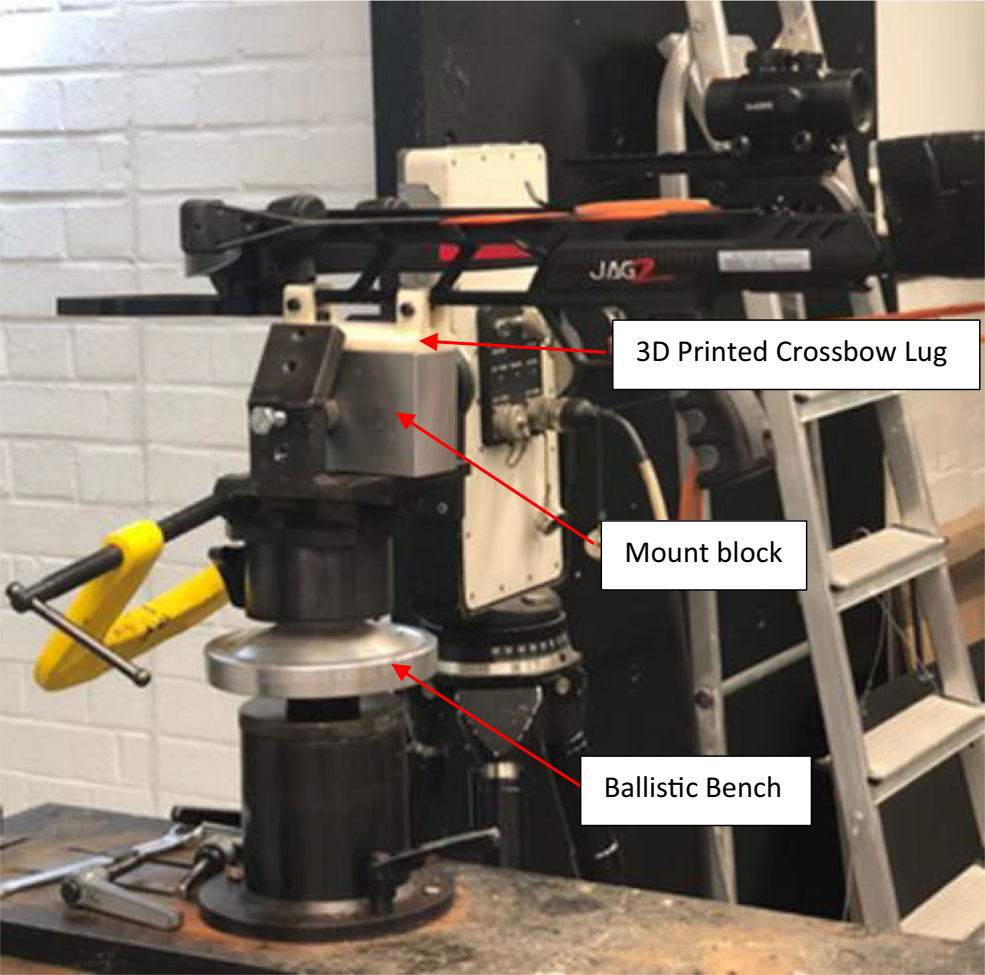
Crossbow mount set up

**Fig. 3:**
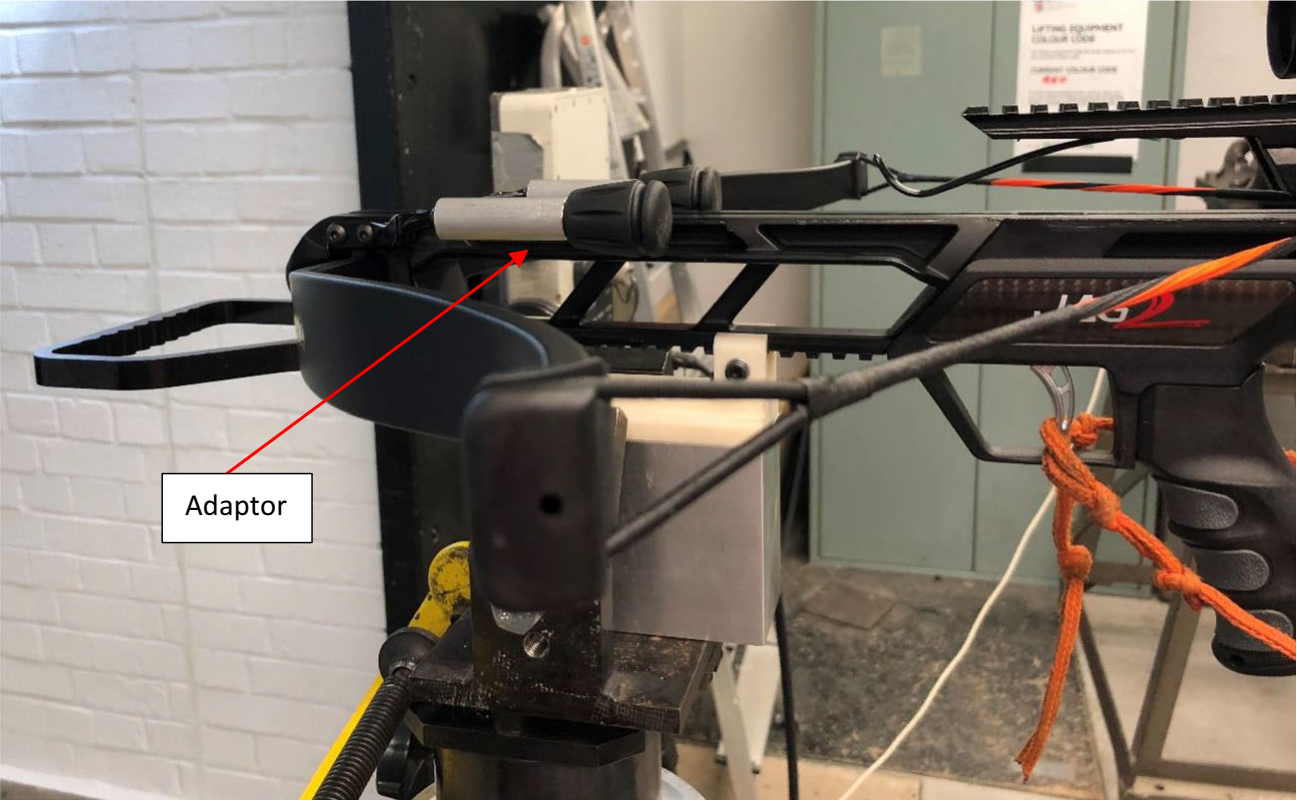
Example of adaptor in place to reduce bolt velocity

**Fig. 4:**
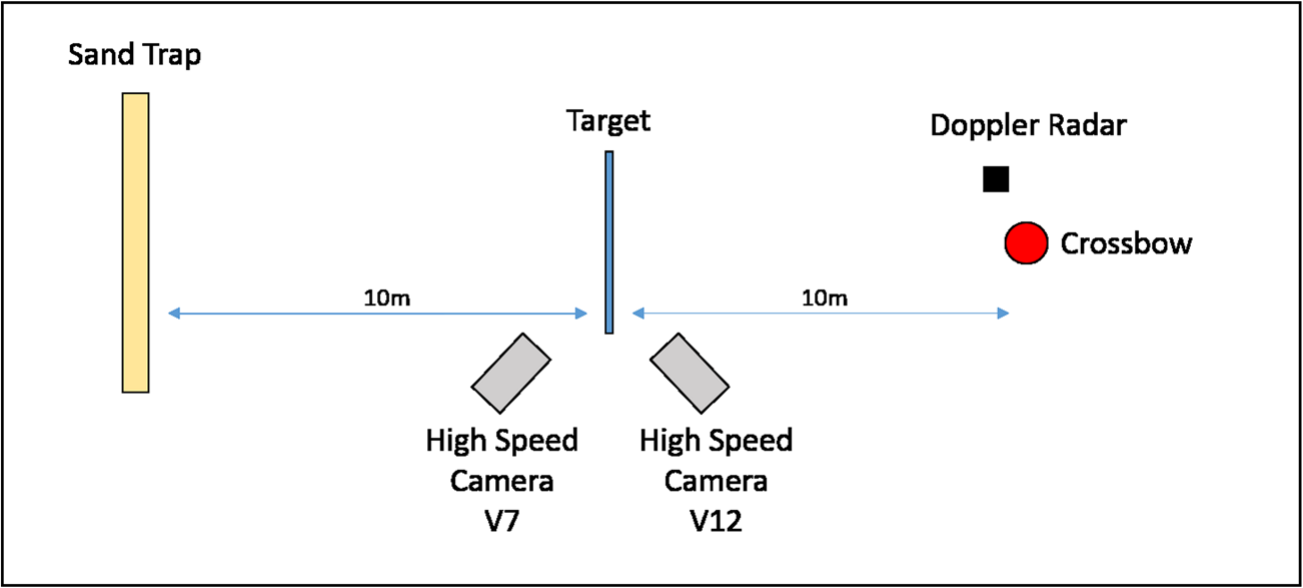
Diagrammatic representation of experimental set up

Testing was conducted at the Small Arms Experimental Range at Cranfield University located at the UK Defence Academy, Shrivenham. The crossbow was mounted in a modified generic firearms mount that consisted of three main components (Fig. 2); a 3D printed crossbow lug, a mount block, and ballistic bench. The firing bench was positioned 1.3 m from the ground and 10 m from the windows which were suspended at 1.40–1.45 m above floor, to allow for a central impact from the fixed crossbow (Fig. 1). A total of six shots were undertaken; three at the maximum velocity of the crossbow (using 2219 bolts) and three at varying velocities (Using EK bolts). To control impact velocity a series of adaptors were attached to the bow at the location of the string stoppers. The test matrix is given in Table 1. The adaptors were engineered to reduce the draw length by 30 mm, 50 mm, and 75 mm (Fig. 3). For each shot, a new bolt was hand loaded and cocked into the mounted crossbow to maintain location constancy. To ensure a central impact on the glass, the crossbow was sighted prior to each shot using the supplied red dot scope, before being remotely fired. In the case of any window perforation, a sand trap located at the end of the range was used to arrest the bolts. Figure 4 shows the layout experimental arrangement.

**Table 1:** Test matrix

Shot number	Bolt type	Adaptor length (mm)
1, 2, 3	2219	0
4, 5, 6	EK	30, 50, 70

### Data acquisition and analysis

To measure impact and exit velocities, Phantom V12 and Phantom V7 high speed cameras with high powered lights were positioned at the front and rear of the window, respectively. High Speed Video (HSV) footage was analysed using Phantom Camera Control software (PCC) 2.6. To obtain velocity, the PCC software was calibrated using a scale to relate pixels to distance. For each video, scale calibration was measured about a known distance (nock length^1^). Radial glass fracture velocity was also calculated using this approach but calibrated using a calibration image with a forensic scale in view.

## Results

**Fig. 5:**
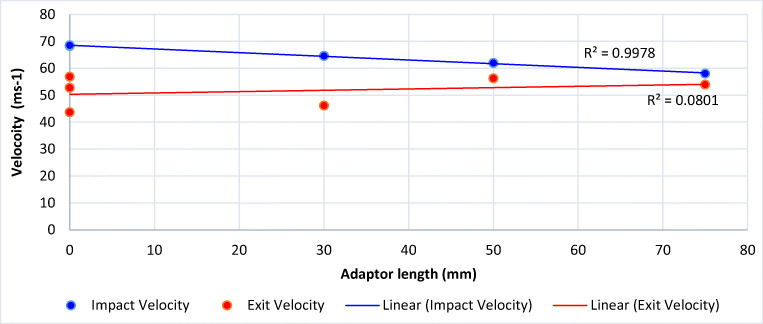
Impact and exit velocity based upon adaptor length

In all tests, the bolts perforated the windows before arresting in the sand trap. Figure 5 shows the raw impact and exit velocities verses adaptor length. With no adaptors, the bolts exhibited mean impact and exit velocities of 68.5 ± 0.17 and 51.1 ± 6.76 m/s, resulting in a velocity drop of 17.4 ± 6.59 m/s. As adaptors were added, bolt velocity decreased linearly (*R*^2^ = 0.99) to 58.1 m/s. Impact velocity did not appear to effect the exit velocity of the bolts, as results were comparable between tests indicating there is no strain rate effect between the velocities studied.

**Fig. 6:**
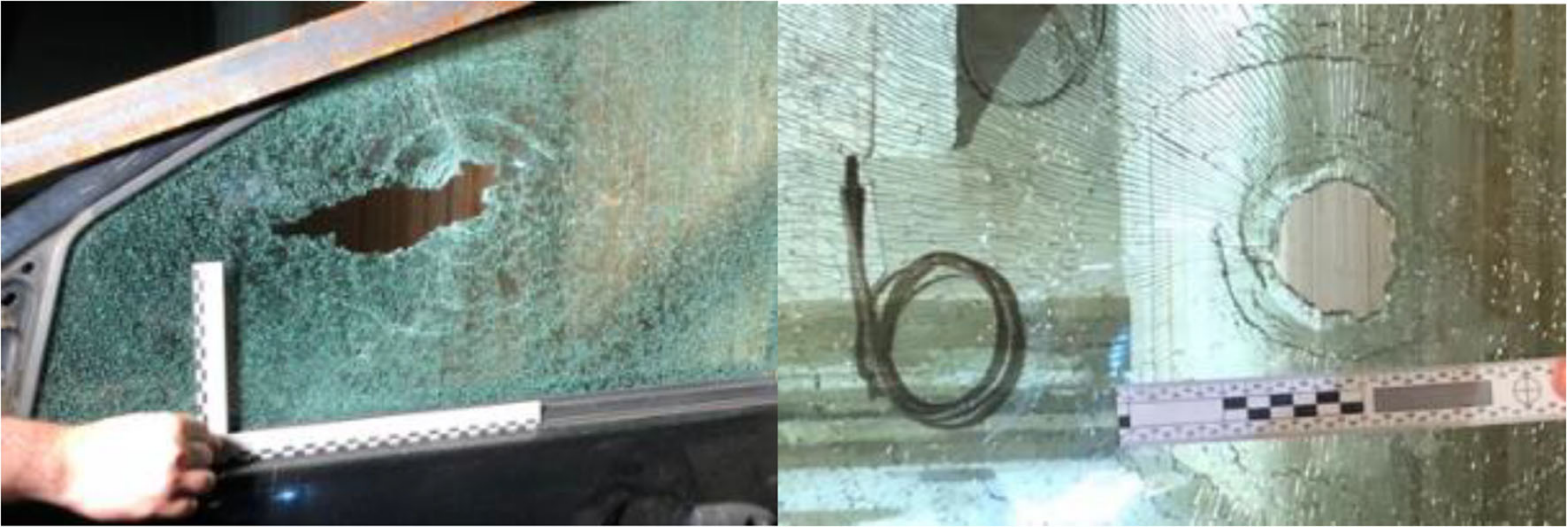
Fracture pattern post impact by a 2219 bolt and EK Bolt in to window 1 and 6, respectively

**Fig. 7:**
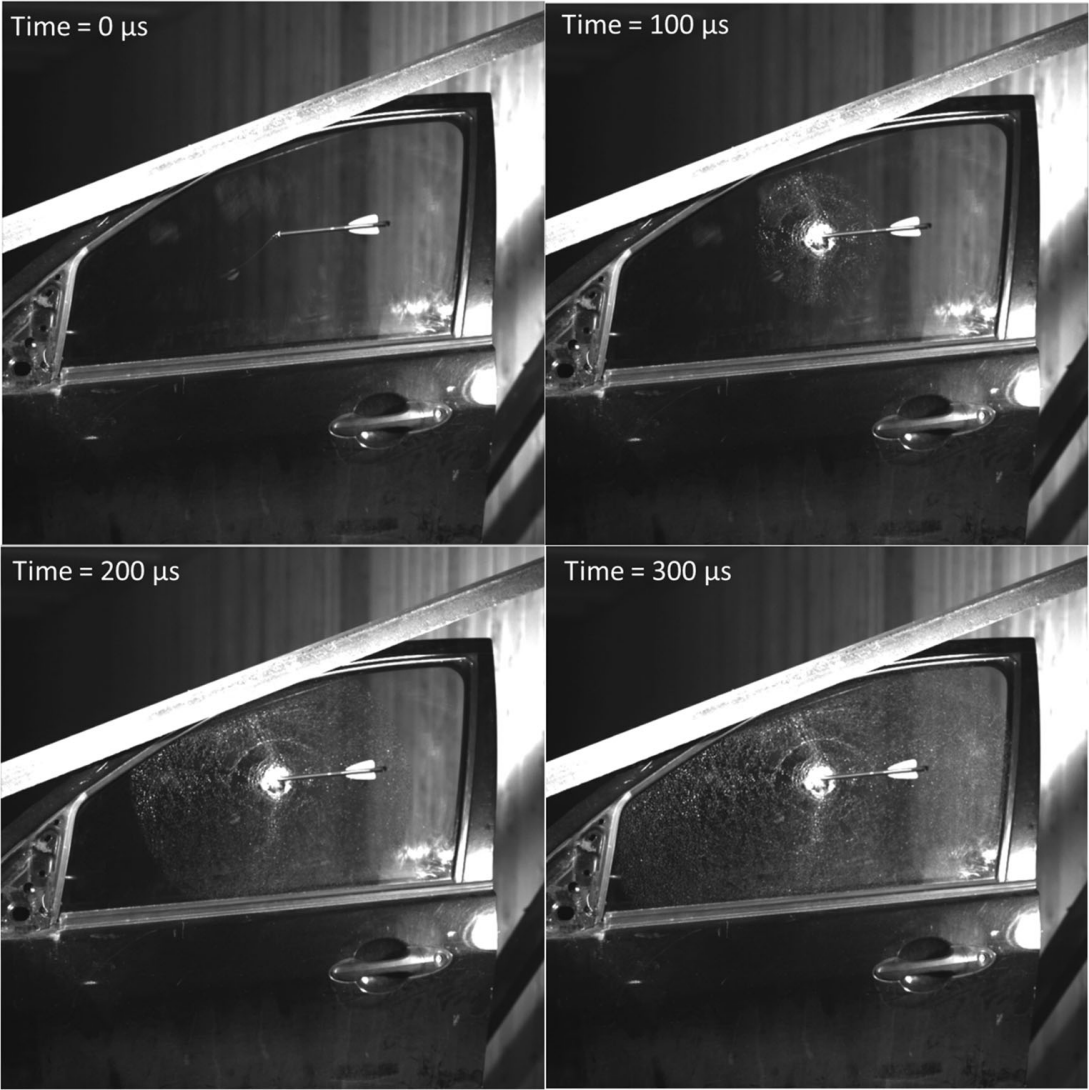
Glass failure during impact with crossbow bolt between 0 to 300 μs during test 1

In all tests, window damage occurred at the point of contact between the bolt and window, resulting in glass shatter (Fig. 6). Damage initiation was radial in geometry, which propagated outwards to the window boundaries at a rate up to 1600 m/s as measured by HSV. At the contact point the glass shards were eroded, leaving a circular hole, whilst the shattered glass remained in place the more distal from the impact. Due to the irregular geometry of the holes five diameter measurements were taken and averaged. Analysis of the circular holes indicated a mean width of 48.6 ± 2.86 mm, 60.9 ± 4.8 mm, and 51.2 ± 4.1 mm for shot numbers 1, 5, and 6, respectively. Raw data is presented in Table 2. No relationship between width and bolt type and velocity was notable. For all other shots, the shattered glass fell away under the mass of the window, resulting in non-measureable holes. Analysis of the HSV found that the local glass erosion was the product of the bolt tip translating through the glass and displaced in a conical pattern (Fig. 7) in the direction of bolt travel.

**Table 2:** Raw glass hole diameter measurements for shots 1, 5, and 6

	Glass hole diameter (mm)		
	Shot 1	Shot 5	Shot 6
1	47.6	65.4	56.9
2	45.9	57.9	48.9
3	51.4	60.7	50.2
4	46.0	65.9	53.8
5	51.8	54.8	46.4
Mean	48.5	60.9	51.2
STD	2.9	4.8	4.1

Impact and post impact damage was also observed in the bolts. Blunting of the tips was observed in all recovered bolt tips (shots 3–6). For the unrecovered tips, the tips and tip holders were missing upon recovery of the bolt. It was shown that following window impact the plastic tip holder separated from the bolt with the tip still firmly attached. In the instance, the tip and holder were retrieved (shot 3), the bonding between the tip holder and bolt had clearly failed. Nock separation was also shown to occur in a similar manner but was limited to the EK research bolts.

Other notable damage was paint removal from the tips. Whilst evident in all recovered tips, it is unclear if it is caused by the window impact or when the bolt enters the sand trap, and thus further investigation is required. Shaft damage was observed only once (shot 4), where the bolt bent approximately 10° 42 mm from the tip. Analysis of the HSV revealed that the damage was caused at the direct point of contact with the window; however, there was no evidence of a non-linear flight, suggesting that such damage could be the product of a manufacturing defect within the shaft.

**Fig. 8:**
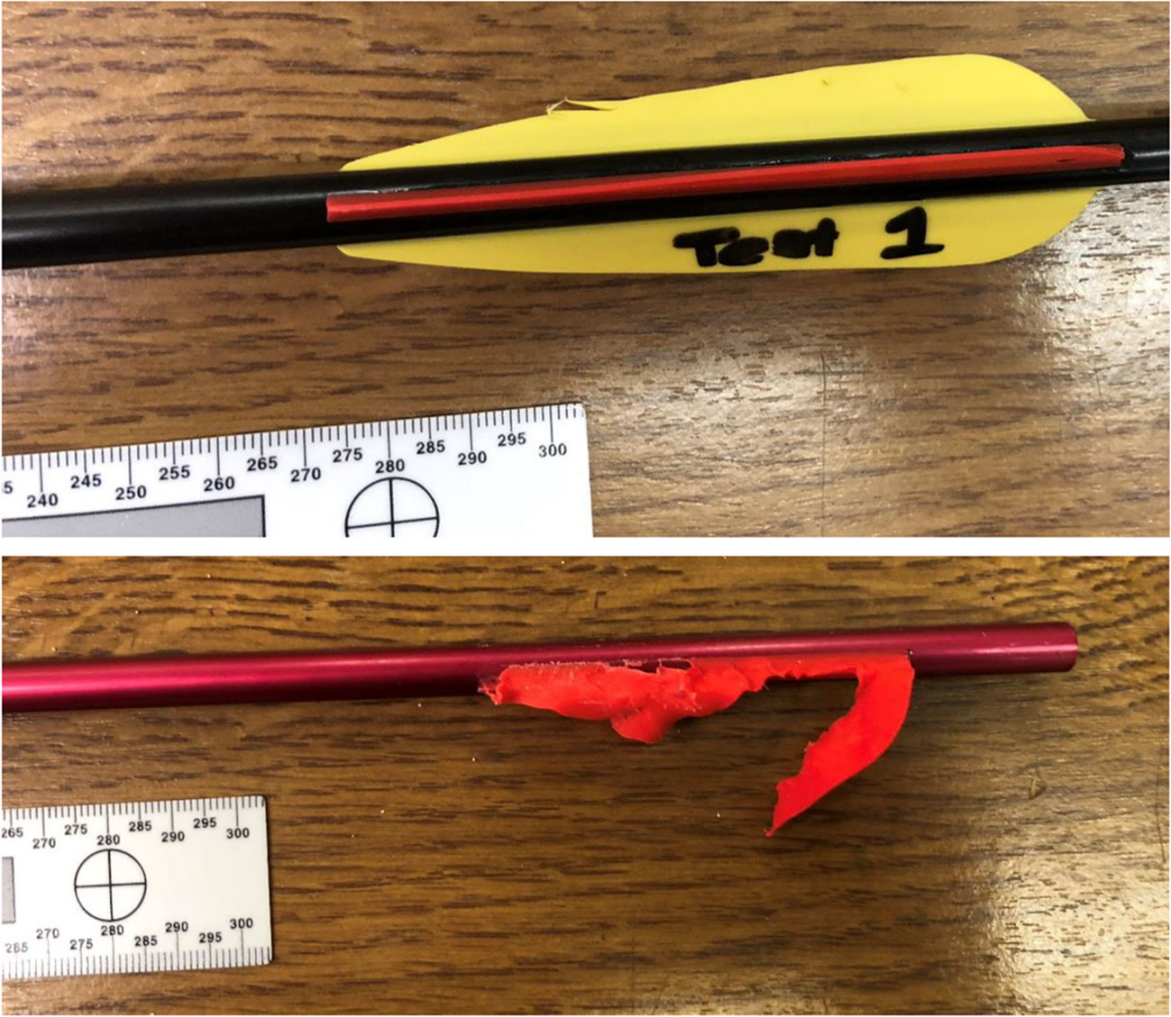
Examples of fletching damage

Fletching damage was the most common with damage ranging from minor tears to traumatic removal (Fig. 8). The cause of this damage is currently unknown; however, HSV suggests that the damage comes from the passing through the windows and interaction with the shattered glass.

## Discussion

Traditional automotive windows have been shown to be an ineffective means of stopping the threat from a closely fired crossbow but offer a potential forensic source post event. In any investigation scene, it is important to understand the potential area where a projectile could land. When the bolts interacted with the windows, there was little evidence to show correlation between impact and exit velocity and bolt type making predicting the final location of the bolt difficult. Nonetheless, if the greatest exit velocity (56.9 m/s) is considered, assuming the bolt has free travel, and no velocity loss is assumed inside the car, the bolt is expected to land within an approximate region of 29 m, based on trajectory mechanics. Should the bolt retain its tip, and penetrate the second car door window (and thus lose an additional 11.7 m/s, assuming no additional velocity loss within the car) the search region will reduce to approximately 23 m. In reality, the true search area will vary as factors including deflection, wind resistance, bolt damage, and skidding will all contribute to the bolts final landing location.

Over the course of testing, it was clear that the majority of bolt damage to the windows result in either the tip, nock, or fletching becoming detached from the bolt, meaning that within a crime scene, these pieces of evidence have potential to be missed if these components end up in secluded locations. Due to the nature of loading a crossbow, these components may contain fingerprint, DNA, and trace evidence such as paint transfer. As HSV highlighted these components detaching from the bolt shaft at the point of contact, the investigator may have to look into a separate location from the bolt to find these components including the car interior. Tip paint removal may also prove to be of significance if it transfers to the surrounding environment. Whilst it is currently unclear if this transfer occurs, HSV indicated that at immediate impact with the window, the bolt tips are blunted whilst post impact analysis showed distinct evidence of paint loss. It is currently unknown if any forensic evidence could be extrapolated from the glass as this was outside the scope of the work.

Window damage failed to be a good indicator of the event or the bolt type used as the failure pattern varied among shots. In the instances that a hole remained within the shattered glass, without the retrieval of the bolt or its subsequent components the event has potential to be confused with a fire arm impact due to the similar failure pattern.

## Conclusions

Two types of crossbow bolts have been tested against a common automotive vehicle where the windows failed to arrest the bolts. No distinct relationship between bolt type, velocity, and window damage was identified; however, bolt damage varied between tests, ranging from tip holder decoupling, shaft damage, and traumatic fletching removal. Prediction of the arresting location of the bolt has been undertaken where the extremes of testing predicted an approximate region of 29 m which reduced down to 23 m following a second window strike. These values however are likely to be effected by factors including deflection, wind resistance, bolt damage, and skidding. As such, further work is required to not only identify the influence of such factors but underpin the findings within this preliminary study due to the limited number of data points reported.

## Footnotes

1 A nock is a notch in the rearmost end of a bolt used to keep the bolt correctly rotated and prevents slipping during the draw or release of the bolt.

## References

[1] BBC (BBC) (2019) Lee Atkins death: murder charge over crossbow death. https://www.bbc.co.uk/news/uk-england-merseyside-48209678. Accessed 24/06/2019 2019

[2] (BBC) BBC (2019) Ilford crossbow killing: Sana Muhammad’s death ‘shocked people to core’. https://www.bbc.co.uk/news/uk-england-london-46210474. Accessed 24/06/2019 2019

[3] (BBC) BBC (2019) Mark Waterfall jailed for Watford GP crossbow shooting. https://www.bbc.co.uk/news/uk-england-beds-bucks-herts-47257772. Accessed 24/06/2019 2019

[4] (BBC) BBC (2019) Liverpool girl, 2, shot in head with crossbow bolt. https://www.bbc.co.uk/news/uk-england-merseyside-48119092. Accessed 24/06/2019 2019

[5] (BBC) BBC (2019) Gerald Corrigan: tributes paid to Holyhead crossbow victim. https://www.bbc.co.uk/news/uk-wales-48241435. Accessed 24/06/2019 2019

[6] Germerott T, Jänisch S, Dieter Tröger H, Günther D (2010). Homicide by bow and arrow. Arch Kriminol.

[7] Eriksson A, Georén B, Öström M (2000). Work-place homicide by bow and arrow. J Forensic Sci.

[8] Hessler C, Hamel W, Kluge S, Mayer U, Grzyska U, Westphal M, Püschel K (2012). Fatal crossbow injury in an adolescent. Arch Kriminol.

[9] Fieseler S, Kunz SN, Peschel O, Zinka B (2011). Suicide with a crossbow. Rechtsmedizin.

[10] Zátopková L, Hejna P (2011). Fatal suicidal crossbow injury-the ability to act. J Forensic Sci.

[11] Jain DK, Aggarwal G, Lubana PS, Moses S (2010). Penetrating craniofacial arrow injury. J Neurosci Rural Pract.

[12] Chang WK, Hsee LC (2010). Crossbow injury in a developed country. Injury.

[13] Smyk D (2009). Crossbow injuries: a case report. J Forensic Legal Med.

[14] Osborne SF, Papchenko T, de Souza CF, Polkinghorne PJ, Hart R (2009). Orbital crossbow injury. Clin Exp Ophthalmol.

[15] Krukemeyer MG, Grellner W, Gehrke G, Koops E, Püschel K (2006). Survived crossbow injuries. Am J Forensic Med Pathol.

[16] Grellner W, Buhmann D, Giese A, Gehrke G, Koops E, Püschel K (2004). Fatal and non-fatal injuries caused by crossbows. Forensic Sci Int.

[17] Karger B, Bratzke H, Graß H, Lasczkowski G, Lessig R, Monticelli F, Wiese J, Zweihoff RF (2004). Crossbow homicides. Int J Legal Med.

[18] Kaye K, Kilgore KP, Grorud C (2004). Transoral crossbow injury: an unusual case of central nervous system foreign body. J Trauma - Injury, Infect Crit Care.

[19] Joly LM, Oswald AM, Disdet M, Raggueneau JL (2002). Difficult endotracheal intubation as a result of penetrating cranio-facial injury by an arrow. Anesth Analg.

[20] Suess O, Kombos T, Suess S, Stendel R, Da Silva C, Brock M (2002). Self-inflicted intracranial injury caused by a crossbow arrow. Eur J Trauma.

[21] Monaghan J (2017) Man who smashed police car windscreen with crossbow bolt to face no further action. https://www.irishnews.com/news/2017/05/17/news/man-who-smashed-police-car-windscreen-with-crossbow-bolt-to-face-no-further-action-1027897/. Accessed 24/06/2019 2019

[22] Ramli J (2017) Seriously injured man wanted over ‘shooting a woman in the leg with a crossbow’ is taken to hospital after being located by police. Mail Online. https://www.dailymail.co.uk/news/article-4727046/Police-hunting-man-involved-crossbow-attack.html. Accessed 2019 2019

[23] The British Association for Shooting and Conservation (2014) The British Association for Shooting and Conservation - Crossbows. The British Association for Shooting and Conservation. P. 2.

[24] Greenwood C (1986) The use and misuse of crossbows. Crossbow Archery Development Association. P. 29

[25] Payne-Gallwey R (2009) Crossbow. Naval and Military Press

[26] Bow Authority Excalibur Matrix Mega 405 – Crossbow Review. Bow Authority. http://bowauthority.com/excalibur-matrix-mega-405-review/. Accessed 21/06/2019 2019

[27] Ballistics101 (2019) 9mm Ballistics Chart. http://www.ballistics101.com. Accessed 13/09/2019 2019

[28] ELong Outdoor (2017) 2219 Hybrid carbon crossbow bolt. http://www.elongoutdoor.com/en/product/2219-Hybrid-Carbon-Crossbow-Bolt.html. Accessed 21/06/2019 2019

[29] Lab BC (2019) EK ARCHERY 20 inch aluminium crossbow bolts. https://www.bushcraftlab.co.uk/products/ek-archery-20-inch-aluminium-crossbow-bolts. Accessed 21/06/2019 2019

[30] Pellpax (2019) EK Archery 175lbs Crossbow Jaguar II. https://m.pellpax.co.uk/archery/crossbows/recurve-crossbow/ek-crossbow-archery-jaguar-ii/17505. Accessed 21/06/2019 2019

